# Video as an alternative to in-person consultations in outpatient renal transplant recipient follow-up: a qualitative study

**DOI:** 10.1186/s12882-021-02284-3

**Published:** 2021-03-22

**Authors:** Cecilie Varsi, Aud-Eldrid Stenehjem, Elin Børøsund, Lise Solberg Nes

**Affiliations:** 1 Department of Digital Health Research, Division of Medicine, Oslo University Hospital, Pb 4950 Nydalen, N-0424 Oslo, Norway; 2 Faculty of Health and Social Sciences, University of South-Eastern Norway, Drammen, Norway; 3 Department of Nephrology, Division of Medicine, Oslo University Hospital, Oslo, Norway; 4 Institute of Clinical Medicine, University of Oslo, Oslo, Norway; 5 Department of Psychiatry & Psychology, Mayo Clinic, Rochester, MN USA

**Keywords:** Kidney diseases, Organ transplantation, Health services, Outpatients, Telemedicine, eHealth, Video consultation, Qualitative research

## Abstract

**Background:**

Renal transplant recipients have to see a nephrologist for regular follow-up for the rest of their lives. To reduce the burden for the patients, video consultation can be an alternative to traditional in-person hospital consultations. The aim of the current study was, from the perspectives of patients and health care providers, to investigate the perceived benefits and challenges of using video consultations in outpatient renal transplant recipient follow-up.

**Methods:**

Patients (i.e., renal transplant recipients; *n* = 18) alternated between regular in-person follow-up consultations and video consultations. Patients and health care providers were then invited to participate in semi-structured interviews. The interviews were analyzed using thematic analysis.

**Results:**

Patients interviewed (*n* = 15) were median 53 years old (range 37–64) and 53% female. The video consultation solution used in the study turned out to have major technical deficiencies. Despite the technical challenges, however, the majority of the patients reported appreciating being able to alternate between video and in-person hospital consultations. Main benefits reported included not needing to travel to the hospital and thereby saving time, less focus on being chronically ill and potential economic benefits for patients and society. The health care providers (*n* = 3) also valued the benefits provided by the use of video consultations, but described the reoccurring technical challenges as disruptive. The fact that patients were in a stable phase of their health condition and already had an established, trusting relationship with their nephrologist, acted as facilitators for success. Possible challenges and harms described included concerns related to security, confidentiality and interruptions, as well as the potential need for physical examinations.

**Conclusions:**

Benefits from using video consultations as an alternative to in-person consultations may outweigh potential technological challenges for patients as well as health care providers. A long-lasting mutually trusting relationship between patient and provider may be an important prerequisite for the experienced benefits of using video consultation. Findings also indicate that starting such care delivery changes in a small-scale, with a few selected patients in a stable phase of their condition, may be an important factor for success.

**Supplementary Information:**

The online version contains supplementary material available at 10.1186/s12882-021-02284-3.

## Background

Transplantation has always been considered the renal replacement treatment of choice, if possible, for patients with end-stage kidney disease [1]. Following transplantation, patients continue to be considered chronically ill as they have to adhere to lifelong immunosuppressive medication regimes and need to pay close attention to all lifestyle choices in order to avoid aggravation of medication side effects and graft dysfunction [2]. The transplantation impacts many aspects of life, including functional status and psychosocial well-being [3], and patients describe life after transplantation as highly demanding and complex, requiring them to manage challenges such as physical symptoms, disability, complex medication regimens, lifestyle adjustments, and having to deal with other consequences such as stress, frustration and fear of transplant failure [3–5].

In Norway, the renal transplant follow-up is organized in the specialist health care service at the hospitals [6, 7]. Renal transplant recipients have to see a nephrologist on regular basis, usually four times a year, when the graft is functioning adequately. In unstable phases, follow-up visits are even more frequent. A few days prior to the consultation, the patients must have blood samples drawn at the hospital in order to have important blood values assessed and discussed in the consultation with the nephrologist. This practice means that the patient has to come to the hospital twice related to each three-month renal transplant follow-up consultation.

Video consultations have been emphasized by health authorities as a potential mean to improve access to care and increase quality of care [8–10]. In order to reduce the disease burden for the renal transplant recipients by decreasing the number of visits at the hospital, video consultation can be an alternative to in-person consultations. A growing body of evidence supports the notion that video consultations may contribute to successful management of chronic illness, including health management following organ transplantation [11–15]. Studies have shown how video consultations are highly appreciated by patients with chronic illness already using such solutions [15–17], that video consultations can enhance engagement and communication between health care providers and patients [15, 16], and that such solutions have been considered to be effective and convenient [14, 17].

Despite the promising findings and potential of video consultations, challenges such as technical problems [18–20] and concerns related to security and privacy [15] have been raised. For example, unstable internet connection has resulted in audio problems and time lag that has interrupted the conversation between patient and provider [18–20]. Some researchers have also argued that video consultations are best suited for consultations where a physical examination of the patient is not required [18, 19] and for settings where an established patient – health care provider relationship is already in place [14].

Renal transplant patients have displayed a positive attitude towards using electronic health (e-health) technology [21]. Video consultation solutions to support renal transplant recipients have yet to be implemented and studied in clinical practice in Norway, however. Therefore, the aim of the current study was to investigate the perceived benefits and challenges of using video consultations in outpatient renal transplant recipient follow-up from the perspective of patients and health care providers.

## Methods

### Study design and participants

The current study explored the patient and health care provider experiences of using video consultations in regular outpatient renal transplant recipient follow-up. The study was conducted at the outpatient clinic at the Department of Nephrology at Oslo University Hospital in Norway, which has regional responsibilities for approximately 50% of the Norwegian population (www.helse-sorost.no). Three health care providers (i.e., one nephrologist and two health support personnel) were requested and consented to participate in the study. As this was the first time video consultations were tested in this particular setting, a decision was made to primarily include renal transplant recipients in stable phases of their transplant trajectory. Purposive sampling was therefore applied to ensure that the patients was in a stable phase, had few expected complications related to their kidney disease, were 18 years or older, were able to speak Norwegian, and have access to their own portable device so that skin changes, oedema and so on could be shown if necessary. Potential participants were identified and informed about the study by the study nephrologist. The study was planned and performed in compliance with the principles outlined in the Declaration of Helsinki [22]. The study was approved by the Oslo University Hospital department for data protection and information security (i.e., Institutional Review Board) (approval number: 18/09405). Written informed consent was obtained from all participants.

### Real-time video consultations

When enrolled in the study, the patients were informed that every second planned three-month follow-up consultations would be conducted by video, unless the patient would be in need of a physical examination at the time. This meant that there would be a video consultation approximately every 6 months for each participating patient.

The video consultation solution Norwegian Health Network (NHN) Cisco meeting application (app), was used in the study. The solution was approved for patient-provider consultations by the hospital department for data protection and information security (i.e., Institutional Review Board equivalent). The NHN Cisco meeting app solution was delivered through NHN specific computer devices in two consultation rooms at the outpatient nephrology clinic. The patients could download and access the Cisco meeting app solution via their own personal computer (PC), tablet or smartphone.

The health care providers received in-person training in how to use the video consultation solution. Security aspects such as being alone in the consultation room during the consultation and also ensuring that the patient is properly identified (i.e., through recognition/social security number) were stressed in the training.

The patients received a letter with information about how to access the solution from their own device, including a personal log-in username and password. The patients were asked to log into the solution a few minutes in advance of the consultation, so that they could be given access to the video consultation as soon as the nephrologist was ready. Patients were also informed about the importance of security precautions and encouraged to make sure they were calling from a private environment where others were not present (i.e., could hear or see them) during the consultations. A phone number was provided for assistance/technical support if needed. The patients were also informed that they needed to go to the hospital to have blood samples drawn a few days ahead of the consultation as well as measure their own weight and blood pressure ahead of the consultation.

### Data collection

Information about patient demographics and illness characteristics, as well as health care provider demographics and work characteristics, was collected through a study specific demographic form at study enrollment (June 2018 to April 2019). Individual interviews were conducted with patients and health care providers 1 year post final participant inclusion (i.e. April 2020). Semi-structured interview guides developed for this study were used, containing questions about the execution of in-person consultations and video consultations, the similarities and differences, the advantages and disadvantages considered, as well as any experienced technical challenges using video consultations (see Additional file 1 for an English version of the interview guide). Interviews with patients were conducted by telephone and interviews with health care providers were conducted in-person at the hospital by members of the research team. The patient interviews lasted 14–34 min. The health personnel interviews lasted 10–20 min. All interviews were audio-recorded and transcribed verbatim.

### Data analysis

Demographics were collected and recorded in Statistical Package for the Social Sciences (release 25, SPSS Inc., Chicago, IL, USA) for simple descriptive analyses. Transcripts were analyzed based on the principles of thematic analysis [23]. In the first step of the analysis, the first author listened to the recordings, read through the transcripts and took notes to become familiarized with the content. Next, using an inductive-deductive approach the first author coded the transcripts into three pre-defined overarching themes based on the interview guide using the software program NVivo version 12 (QSR International, Victoria, Australia). The overarching themes included: 1) In-person consultation execution, 2) Video consultation execution, including technical challenges, and 3) Comparisons between in-person and video consultations, including similarities and differences, advantages and disadvantages. Sub-themes were identified as they emerged under each theme, and the transcripts were coded into these sub-themes. Each theme was then re-examined, identifying variations and similarities within the themes and sub-themes. The co-authors then discussed and reviewed the themes and sub-themes and subsequently renamed and re-arranged them into a final structure.

## Results

### Participant information

Seventeen renal transplant recipients, 1 patient in pre-transplant phase, and 3 of their health care providers were included in the study and used video consultations in outpatient follow-up. Patient participants were enrolled in the study for minimum 12 months (June 2018 – April 2020). Patients’ self-reported number of video consultations undertaken during the study period ranged from one to ten.

Of the 21 participants enrolled in the study, 15 patients and all 3 health care personnel agreed to participate in the post study interviews. Of the three patient participants not consenting to participate in the interviews, one had never conducted a video consultation and two did not give a reason for declining.

Of the patients (*n* = 15) participating in the interviews, gender was equally represented (female 53%), age was median 53 years old (range 37–64), most were married or cohabitating (12/15, 80%) and all except one were renal transplant recipients. Most of the participants (12/15, 80%) owned a smartphone, and rated their digital user experience (i.e., PC/tablet/smartphone use) as high. Please see Table 1 for demographics and illness characteristics for the patients participating in the interviews).

**Table 1:** Baseline demographic and illness characteristics for patients participating in interviews (*n* = 15)

Characteristics	Value
**Age (years)**, median (range)	53 (37–64)
**Gender**, n (%)	
Female	8 (53)
Male	7 (47)
**Marital status**, n (%)	
Married/cohabitating	12 (80)
Single/divorced	3 (20)
**Education**, n (%)	
Elementary/high school	8 (53)
University/college < 4 years	4 (27)
University/college > 4 years	3 (20)
**Employment**, n (%)	
Full-time/part-time work	10 (67)
Sick leave/disability benefits	5 (33)
**Years since kidney related diagnosis**^**a**^, median (range)	29 (5–45)
**Renal transplantation**, n (%)	14 (93)
**Years since renal transplantation**, median (range) (*n* = 13 ^b^)	15 (4–26)
**Second renal transplantation**, n (%)	2 (13)
**Years since second renal transplantation,** median (range) (*n* = 2)	18.5 (14–23)
**Owns a PC,** n (%)	9 (33)
**Owns a tablet**, n (%)	6 (40)
**Owns a smart phone**, n (%)	12 (80)
**User experience:** (Scale 0 = low to 5 = high), median (range)	
PC	4.5 (1–5)
Tablet	4.0 (1–5)
Smartphone	5.0 (3–5)

^a^Diagnosis includes: polycystic kidney disease, hypertension/nephrosclerosis, chronic glomerulonephritis, chronic nephritis. ^b^Missing data from one participant as well as one participant not yet having received transplantation

The participating health care providers (*n* = 3) were female. All three had access to their own PC, tablet and smartphone, and rated their user experience as very high (median 5).

### Overview

**Fig. 1:**
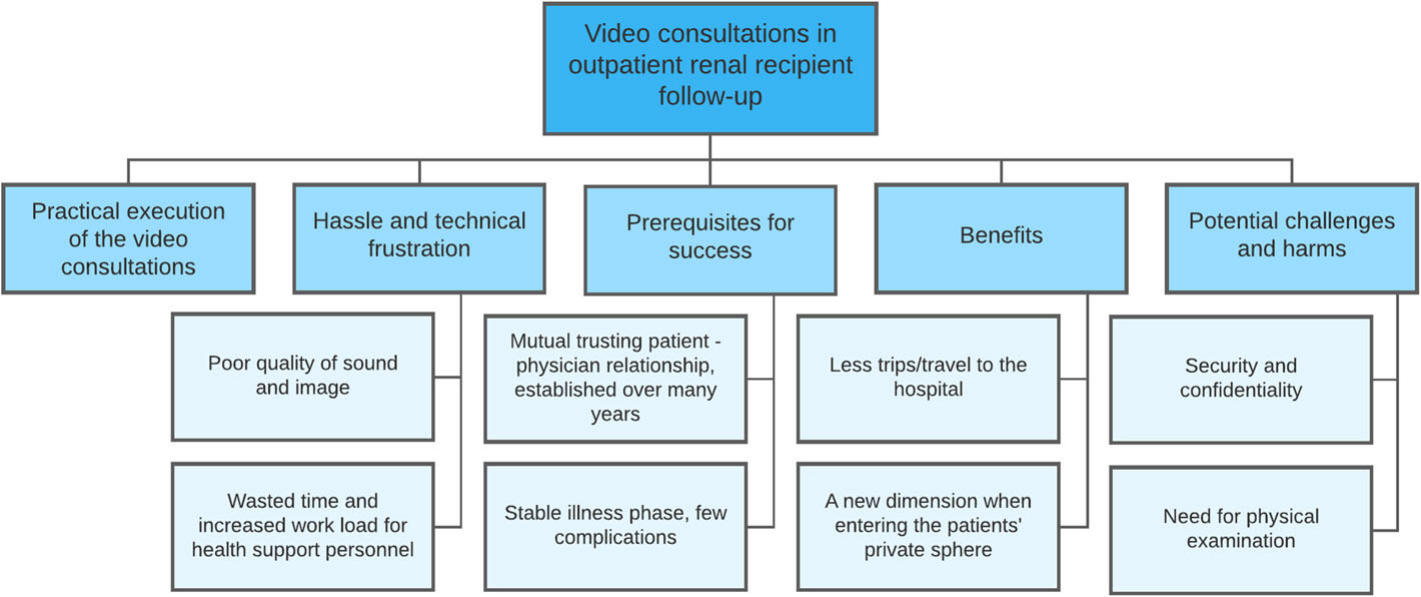
Overview of main themes and sub-themes

Transcripts from the interviews were analyzed into five main themes: (1) Practical execution of the video consultations, (2) Hassle and technical frustration, (3) Prerequisites for success, (4) Benefits, and (5) Potential challenges and harms. Main themes and sub-themes are illustrated in Fig. 1.

### Practical execution of the video consultations

The alternating between in-person and video consultations was considered an appropriate arrangement by patients as well as the nephrologist. The majority of the patients interviewed said the information received in advance about how to download and use the video solution was sufficient to get them started. However, some of the patients expressed concerns regarding the procedure, stating that it might be too difficult to follow for people with less technical knowledge/experience, and suggested that the hospital staff could provide more direct “get started” help for those who might need it. One of the patients said:

So maybe finding out in advance how much the person knows about the technical things. Maybe that could be a good idea. Or maybe say that you will get help from someone to do this. But I think maybe it would have been nice and reassuring, and good service, if you had time to show how it should be done. (Patient 1)

Half of the patients reported conducting the video consultations from home, and half of them from work. The latter was possible when the patients had a private room available at work where they could close the door to participate in the consultation.

A majority of the patients reported using their smartphone for the video consultations, closely followed by those using tablet or PC. Two of the patients bought a new device due to lack of compatibility with the NHN Cisco meeting app video consultation solution.

### Hassle and technical frustration

Almost all of the patients interviewed had experienced technical challenges using the video consultation solution. One of them reported having had to stop using video consultations and go back to in-person consultations at the outpatient clinic due to technical issues/not getting the equipment to work.

#### Poor quality of sound and image

The problems experienced related to sound and image were multifaceted. Patients reported sometimes being unable to get in touch with the nephrologist through the video consultation solution. Other times the sound had been of bad quality (e.g. choppy) or had totally disappeared. Patients also reported that the images sometimes disappeared and sometimes froze. Many of the patients also described having experienced new types of technical problems from video consultation to video consultation. One of the patients said:

It was probably not fully synchronized, sound and image. And once there was a picture, without sound. And then there was sound, without image. (Patient 13)

The patients and health care providers stated that it had not always been possible to detect the cause of the error, but in many cases there were concrete reasons for the errors. For example, video consultations where the patients were at work could be hindered by network fire-walls. One error that took the team some time to discover, was that the video solution went into “sleep mode” if the patient logged into the video solution too long before the consultation started. One of the health support personnel explained:

So we found out lately, the patient has been in the app for too long, so the app has gone to sleep. So then they [the patients] have to restart the app, and then we usually get the connection going. (Health support personnel 1)

Also, some user errors caused by the patients themselves were reported. For example, the patients lost their password or they bought a new telephone or computer and were unable to download the application onto the new device. Also, there were some errors related to that the patients had not allowed the solution to use the computer microphone. Patients as well as health support personnel reported having spent significant amount of time to make the video consultation solution work. Patients and health care providers alike reported that many patients had needed help with downloading and start-up of the solution.

Even if most of the patients had experienced technical problems during their video consultations, there was a collective understanding that start-up problems when using such new solutions were quite usual. One of the patients said:

There was a technical failure in the beginning, and then we were up and running again. That is to be expected in the beginning. This is after all a pioneering project, I think, at least for me. So I guess some hassle in the beginning is to be expected. (Patient 3)

#### Wasted time and increased work load for health support personnel

In case of technical problems, the health support personnel in the current study had to be available for technical support during each video consultation. As such, they experienced increased work load and was occupied for as long as the consultations lasted. One of the health support personnel said:

I have tried to be in the doctor's office every time there was a video consultation. If I have not been there, and the doctor has not been able to get in touch with the patient, then the doctor must first go to find me, I must come to the consultation room, and then we find the patient's cell phone number, and then I have to call the patient. So it can take a lot of time. (Health support personnel 1)

More than half of the patients described having had to continue the consultation by telephone one or more times, after trying and failing for a while when technical problems occurred. One of the patients stated that every consultation had started with video and ended by telephone:

It has actually been so bad that in the end we have decided to do the rest of the consultation by phone. (Patient 12)

### Prerequisites for success

The interviews revealed that there were some elements that acted as prerequisites for the overall positive attitude towards video consultations, despite the apparent pervasive and comprehensive technical problems.

#### Mutual trusting patient – physician relationship, established over many years

Half of the patients reported having had the same nephrologist, without exception, during their respectively 3–25 years follow-up at the hospital. Three of the patients had had follow-up by other nephrologists for some years, and five of the patients had had other nephrologists only when their usual nephrologist was on vacation or for other reasons unavailable. As such, the patients in the current study had an already established, long-term trusting relationship with their nephrologist. In that regard, the patients stated that changing the consultation from in-person to video did not represent too much of a change for them as the consultation followed the same structure and covered the same topics whether they were in-person or by video. One patient said:

It's the same. The difference is probably only that I'm physically there. We do the same things. [...] And I do not feel that there’s any more security in being with the physician in-person, as opposed to being at home. I feel as confident and safe with the treatment and what we agree on by video, as what I do when present in the doctor's office. (Patient 6)

The nephrologist also acknowledged the video consultation as equivalent to the in-person consultation:

For the doctor, I would say it is the same. We have our appointment schedule, and that time is set aside for each patient. Whether it's in-person attendance, or by video, it really does not matter. (Nephrologist)

#### Stable illness phase, few complications

All patients in the study were in a stable phase of their kidney disease. Medical challenges and issues were stable and well monitored, and there were no need for physical examinations. Some of the patients did however express need for re-assurance that they could ask for an in-person consultation if they felt it was necessary.

### Benefits

The majority of the patients interviewed stated that they wanted to continue with video consultations, and would also recommend them to others, even if they had experienced a lot of technical problems during the study. The health care providers also stated that the benefits with video consultations outweighed the problems. One of the health support personnel summarized:

I would like to emphasize that despite the fact that we have experienced a number of challenges, we have been very satisfied with the video consultations, when the solution has worked. The fact that it has worked has outweighed all the challenges we have faced, because it is so much fun when it actually works, because it is such a good offer. And from what I have seen from the patients, who have used video consultation, they are also very satisfied when it actually works. (Health support personnel 1)

#### Less trips/travel to the hospital

The benefit most often reported by patients as well as health care providers was the time saved and that the patients did not have to travel to the hospital for the consultations. As such, the video consultations were viewed as effective, practical and convenient. One patient said:

This has meant that I don’t have to set aside so much time. I have to go all the way to the hospital. It takes, as I said, three to four hours, back and forth, and so on. So I can do it in ten minutes, which means that I have time to do other things instead, or plan other things. So for me it has been absolutely superb. (Patient 3).

Patients with long as well as short travel distance to the hospital reported appreciating that they did not have to go to the hospital. This was especially the case on days when they did not feel well, and for persons with physical disabilities. They reported appreciating still having the option to conduct the consultation, not needing to cancel if they did not feel well enough to travel. Some of the patients also mentioned the economical aspect, that money usually spent on travel was saved and also that there was a socio-economic aspect of not needing to leave the workplace to go to the hospital for consultation. Some patients also mentioned psychological benefits related to the use of video consultations. For example, one of the patients reported feeling less stressed when conducting the consultation from home, as visits to the hospital usually came with feelings of stress and anxiety. Another patient described experiencing fewer burdens with chronic illness:

But I think that being able to have the consultation by video is much, much better, because you do not have to [...] spend time, and travel, and leave work. When you, somehow, do not want to be chronically ill, as it is called. So it's okay to have minimal absence due to consultations then, since I do it every three months. It's not much, but when you've done it for 20 years, it's a lot. So for me, it's a huge, huge improvement that I can do it [by video]. (Patient 8)

As the interviews were conducted right around the beginning of the Covid-19 pandemic situation, several of the patients mentioned that the pandemic situation, with the risk of disease spreading had strengthened their positive attitude towards video consultations. One of the patients said:

It is one of the advantages, when such situations occur [Covid-19], that you can then still keep in touch with the doctor. Especially for us who are especially at risk. So if I was to mention an extra benefit when you have diseases [in the surroundings] that are contagious, that makes a video consultation beneficial [because you can stay at home and decrease your own risk of being infected]. (Patient 5)

#### A new dimension when entering the patients’ private sphere

The nephrologist reported experiencing that entering the private sphere of the patients, when the video consultations brought the nephrologist into the patients’ homes, was an unanticipated positive experience:

Actually kind of interesting too, because then you are in their homes, or you are at their cabin, or wherever they are. So you kind of get more, an additional dimension, in the doctor-patient relationship, which I think has been rather rewarding. (Nephrologist)

### Potential challenges and harms

Some patients said they could not think of any disadvantages to using video consultations, while some described how they thought video consultations could carry the potential for harm. Patients did not describe potential harm as personal experiences, but rather as elements they imagined as potential challenges and threats for harm.

#### Security and confidentiality

Most patients did not mention any aspect of security or confidentiality. Only one patient mentioned a fear of leaking of sensitive information, or getting hacked by unauthorized persons. One patient expressed trust regarding own personal information, and trusted the storing and log-in routines of the video solution chosen by the hospital for this study. Also, one patient said it was important that the routines from the hospital were followed so that there were no other persons in the room together with the physician, without the patient being informed about it. Some patients mentioned that it could be difficult to find a quiet place for the video consultation from work or from home, for example if they were home together with family members. One patient said:

So I don’t think I would have liked, even if it may be my spouse, I would not have liked to carry it out if the spouse was sitting in the living room, or in the same room as me. (Patient 7).

#### Need for physical examination

Several of the patients mentioned that there was a potential risk of overseeing important signs and symptoms when the consultation was conducted over video and not in-person, particularly related to aspects such as pain, wounds, weight, blood pressure, heart rate and so on. One of the patients said:

The downside, of course, is that there may be things that you are not aware of, that the doctor can discover physically, when in-person. [...] There is an extra safety when the doctor performs physical examinations, than when you perform those examinations on yourself. (Patient 5).

The nephrologist also described the potential risks of overseeing important aspects:

If there were clinical findings, which in a way, will not be discovered, because you cannot examine the patient. It will only be visual and auditory, right. You don’t get the third dimension, the physical examination. (Nephrologist)

## Discussion

The current study examined the experiences of renal transplant recipients and health care providers when using video consultations in follow-up after renal transplantation. The respondents interviewed in this study described the video consultation solution used as having major technical deficiencies, including connection problems and poor quality of sound and images. Despite the technical challenges experienced the majority of the patients appreciated being able to have video consultations as an alternative to in-person consultations at the hospital. The main benefit reported by patients was that they did not need to travel to the hospital, stating that this meant time savings, less focus on being chronically ill and economic benefits for patients as well as the society. Health care providers also described appreciating the benefits involved in using video consultations, but did describe significant disturbances experienced by the technical challenges. Facilitators for succeeding with video consultations included the patients being in a stable phase of their disease, and already having an established, trusting relationship with their nephrologist. Potential challenges and harms of video consultations were described as most likely related to security, confidentiality, interruptions and if there should be a need for a physical examination by the physician.

### The benefits outweighed the technological shortcomings

The patients in the current study described a number of benefits related to using video consultations. Time savings was one of the benefits reported, with patients living far away saving a significant amount of time. However, patients living or working near the hospital also appreciated the benefit of time saved. This is in line with previous studies showing that time spent on travels is unwelcome to patients [14, 17, 24]. As renal transplant patients have to have routine follow-up for the rest of their lives, being able to have some of these follow-up consultations (i.e., every second follow-up in the current study) via video appeared to bring a sense of freedom to the patients. Traditionally, the routine in-patient consultations at the hospital were set, but with the new alternative, patients could attend the consultation from wherever they wanted, which provided them with a new sense of freedom and flexibility. The patients could even travel around the world if they wanted to, and still be able to have close follow-up from health care providers that they knew and trusted at home.

Technological options and solutions are constantly evolving, also within healthcare. Challenges with and disruptions from the technology frequently occur however [14, 18–20], and in the current study, there was significant technological hassle with the video consultation solution used. Research has pointed to the need to avoid technological problems as much as possible, as patients receiving health care are in a vulnerable situation and may lack the energy to keep trying if the technology does not work [25]. In the current study, however, patients continued to be motivated for use, even if the technology presented with significant issues, and patients as well as health care providers remained positive towards the use of video consultations despite these issues. One reason for this might be that many of the patients were experienced technology users. Another factor could be that the participating patients in the current study were in a stable phase of their disease, with only limited disease related concerns. Perceived seriousness of the conditions has also been seen to be a key factor influencing patients’ willingness to use electronic consultations, as lower use has been associated with patients in poorer health conditions [26].

In line with previous studies [17, 24] the patients in the current study indicated that they saved money by attending video consultations compared to in-person hospital consultations. In contrast, however, video consultations such as the ones tested in the current study save no time or money for the health care providers or the hospital itself. The physicians may spend the same time on the consultations, regardless of type (i.e., in-person or video), but in the current study the health support personnel spent more time on the video consultations, trying to solve technical issues. Such challenges may even lead to a need for more preparations in advance of, and follow-up during, consultations. Such added aspects of consultation routines will create staff-related as well as financial challenges. Reimbursement, or lack of adequate reimbursement systems, is described as another major barrier to the implementation of video consultations [27]. If the reimbursement systems are not in place, providing options for reimbursements equivalent to that of in-person consultations, electronic/video consultations will never reach implementation potential. The occurrence of the Covid-19 pandemic, although detrimental, has however triggered an urgent need for implementation of video consultations in patient follow up, as video consultations can be a practical solution for many chronic conditions or during unusual health care challenges and situations such as the current Covid-19 pandemic. This might especially be helpful for people with a high risk for hospital infections, for families with chronically ill children or less mobile patients, for whom traveling to the outpatient clinic is burdensome or for those whose therapy data are already available in software clouds. Furthermore, the reimbursement barriers are slowly being addressed [28]. However, as reimbursement for video consultations cannot surpass that of in-person consultations, the implementation of such solutions depends on reliable, easy to use technical solutions that require minimum technical support. In addition, new and efficient systems for support need to be established in order for health care providers to be allowed to do their job of providing care, rather than technical support. Supporting existing research [14, 15, 29], the current study demonstrates that video consultation implementation needs to be prioritized in order for it to be incorporated as a fluent part of clinical practice.

### Aspects of human interaction

The results from the current study underlined an established, trusting relationship between patient and physician as a prerequisite for the video consultations to be experienced as a good service. An established, mutually trusting relationship has also previously been demonstrated by a number of studies as a key factor for success [30]. The current study further emphasized that not only do the patients need to trust their nephrologist, but the nephrologist must also trust the patients. In the current study, the mutually trusting relationship allowed the patients to trust that they would receive the same high quality of care, whether they met the nephrologist in-person or by video. Likewise, the nephrologist had to trust that the patients would be able to measure and report important physical changes, for example occurrence of rashes or edema, to the nephrologist. Findings show that that some of the responsibilities that have traditionally had been placed within the health care service, and for renal transplant follow-up especially on the nephrologist, can be transferred to the patients. The patients in this study had to measure physical functions, such as blood pressure and weight, usually measured by health care providers at the hospital, by themselves. Whether delivered in-person or by video, the post renal transplant follow-up consultations are very much the same, as described by patients as well as health care providers in this study. This can likely be explained by the routine follow-up nature of the consultations (e.g., brief and precise, focusing mainly on kidney/graft function and blood pressure). As such, the results from the current study showed renal transplant follow-up as suitable for video consultations, particularly when providing patients and health care providers alike with the opportunity to alternate between in-person and video follow-up.

The use of video consultations may not be suitable for all types of patients, not even all types of patients post renal transplant. Are the patients capable of conducting the measures needed for the physician to be assured of their health situation? Can adequate care be provided should there be the need for a physical examination? Renal transplant recipients may also feel isolated after the transplantation, with the contact with the hospital being considerably reduced [31]. Patients’ emotional and support needs should not be overlooked [31, 32] but may be more challenging to detect and address when not meeting the patient in-person.

Despite the many potential benefits with the use of video consultations, aspects of appropriateness and professional soundness have to be carefully considered and evaluated before replacing or offering alternatives to in-person consultations. Individual considerations are required.

### Strengths and limitations

There are number of limitations to this study. First, the study was conducted at a single university hospital, and the results may not be representative for other practice settings. However, the outpatient clinic in which the study was conducted is one of the largest Nephrology clinics in Norway, with regional responsibility for half the country’s population, and all results are annually reported to the national quality register [6]. Second, the study included a limited number of patients and health care providers. However, even though the sample is small, the data material presents with a significant amount of informative input and knowledge that may be important to improve future health care settings. As the current study aimed to gather rich and varied data, this means that statements from one participant could be as important as statements of the majority. Furthermore, the small sample size ensured that all procedures could be well tested and further refined before providing a larger population of patients and health care providers with the option of video consultations. Third, all patients included in the current study were identified and selected by the nephrologist to make sure that the participants were in a stable phase of their disease and also somewhat familiar with technology. This could have introduced a selection bias and as such be considered a limitation, but it also ensured professional soundness (i.e., stable phase of the disease) and made it possible to conduct the video consultations despite the many technical challenges presented by the video solution in use. Finally, the interviews conducted in the current study provided insight into a variety of patient experiences and as such allowed sufficient depth in the analyses, which can be considered a strength.

### Future implications

Future studies examining the use of video consultations in renal transplant recipient follow-up should aim to include larger patient samples from multiple centers and strive to incorporate more variation in terms of where the patients are in the disease trajectory. This could involve including patients in less stable phases of their disease, if considered appropriate, justifiable and professional sound to do so. Furthermore, future options to have health data such as blood pressure, weight, body composition, heart frequency or oxygen saturation transferred by apps directly to the Health care team or into a personal health care suite available in advance of the video consultation should also be explored. When scaling up to larger studies comprising more patients as well as health care providers, the aspects of implementation (e.g., how to best implement new types of follow-up, such as video consultations, into routine care) should also be addressed [29].

## Conclusions

The current study offers insight into the use of video consultations in outpatient renal transplant follow-up from the perspective of patients and health care providers. The results indicate that the benefits experienced by patients as well as health care providers surpassed any technological challenges encountered. An important prerequisite for the experienced benefit was a long-lasting mutual trusting relationship between patient and physician. Findings also support the notion that initial small-scale studies, with few selected patients in stable phases of their condition, may lay the groundwork before providing a larger population of patients and health care providers with the option of video consultations.

## Supplementary Information

**Additional file 1.** English version of the interview guide

## Data Availability

The datasets generated and analyzed during the current study are not publicly available due to national ethical regulations, but can be available from the corresponding author on specific request, if approved by the hospital department for data protection and information security (i.e., Institutional Review Board equivalent) at Oslo University Hospital.
