# Supplementary material for: Video as an alternative to in-person consultations in outpatient renal transplant recipient follow-up: a qualitative study
Source: BMC Nephrol. 2021 Mar 22;22:105. doi: 10.1186/s12882-021-02284-3 (PMC7983085; doi:10.1186/s12882-021-02284-3)
Supplement: Supplementary file 1 — Additional file 1. English version of the interview guide [file 12882_2021_2284_MOESM1_ESM.zip › 2020-12-16 Interview guide HCP.docx]

# Additional file 2

# Semi-structured interview guide for interviews with health care providers

## Introduction

*Study personnel give a brief introduction about the aim of the study and the topics to be discussed in this interview.*

## The renal transplant follow-up

Can you tell me about how a typical renal transplant follow-up consultation at the outpatient clinic is conducted? What is the difference between an in-person consultation and a video consultation? Can you describe your role in the video consultation project?

## Accomplishment of video consultations

Can you tell me about the routines for the accomplishment of video consultations? How did you experience having dialogue via video? Were there issues you did not want to address on video? How much time did you spend on video consultations compared to in-person?

## Technical aspects

How was the quality of the technical solution, including sound and image? Was there any technical problems, and if yes, how were they solved? Did you need to cancel any of the video consultations due to technical problems? Was it difficult to understand what the patient said? Was there any misunderstandings? Can you tell me about the training and support you received?

## Advantages and disadvantages

Can you tell me about advantages using video consultations instead of in-person consultations, from the provider, service and patient perspective? Do you see any disadvantages? Will you recommend video consultations to your colleagues? Which patient groups do you think video consultations are suitable/unsuitable for? Have you experienced any unforeseen events when using video consultations?

## Closing remarks

*Thank the participant for the insight shared and time spent during the interview.*
