# Supplementary material for: Video as an alternative to in-person consultations in outpatient renal transplant recipient follow-up: a qualitative study
Source: BMC Nephrol. 2021 Mar 22;22:105. doi: 10.1186/s12882-021-02284-3 (PMC7983085; doi:10.1186/s12882-021-02284-3)
Supplement: Supplementary file 1 — Additional file 1. English version of the interview guide [file 12882_2021_2284_MOESM1_ESM.zip › 2020-12-16 Interview guide patients.docx]

# Additional file 1

# Semi-structured interview guide for interviews with patients

## Introduction

*Study personnel give a brief introduction about the aim of the study and the topics to be discussed in this interview.*

## The renal transplant follow-up

Can you tell me about how a typical renal transplant follow-up consultation at the outpatient clinic is conducted? For how long have you been followed up for your kidney disease? How many nephrologists have you sees during the follow up trajectory? What is the difference between an in-person consultation and a video consultation? How much time do you spend on video consultation compared to in-person consultation, including preparations and travel time? Can you tell me about the measurements you need to do in advance of the consultation, such as body weight, blood pressure, blood samples etc. What is your experience with taking care of these measurements yourself?

## Accomplishment of video consultations

Can you tell me about the routines for the accomplishment of video consultations? How was the consultation arranged and how did you log on to the system and get in touch with the nephrologist? How many video consultations have you had? How did you experience having dialogue via video? Were there issues you did not want to address via video? How much time did you spend on video consultations compared to in-person consultations? Can you describe the presence and time available from the nephrologist? Where did you stay when having video consultations (home, work, other)?

## Technical aspects

Which device did you use (computer, tablet, smart phone)? How was the quality of the technical solution, including sound and image? Was there any technical problems, and if yes, how were they solved? Did you need to cancel any of the video consultations due to technical problems? Was it difficult to understand what the nephrologist said? Were there any misunderstandings? Can you tell me about the training and support you received?

## Advantages and disadvantages

Can you tell me about eventual advantages using video consultations instead of in-person consultations? Did you experience any disadvantages? What has video consultations meant for you? Will you recommend video consultations to other patients? Is there anyone you think video consultations are more or less suitable/unsuitable for? Have you experienced any unforeseen events when using video consultations?

## Closing remarks

*Thank the participant for the insight shared and time spent during the interview.*
